# Pentamethylquercetin Improves Adiponectin Expression in Differentiated 3T3-L1 Cells via a Mechanism that Implicates PPARγ together with TNF-α and IL-6

**DOI:** 10.3390/molecules16075754

**Published:** 2011-07-06

**Authors:** Lei Chen, Ting He, Yi Han, Ji-Zhong Sheng, Si Jin, Man-Wen Jin

**Affiliations:** Department of Pharmacology, Tongji Medical College, Huazhong University of Science and Technology, Wuhan 430030, China; Email: clean829940@sina.com (L.C.)

**Keywords:** adiponectin, pentamethylquercetin (PMQ), 3T3-L1 adipocytes, PPARγ, TNF-α, IL-6

## Abstract

Adiponectin is an adipocyte-derived hormone that plays a pivotal role in the regulation of lipid and glucose metabolism. Up-regulation of adiponectin expression and production has been shown to benefit for metabolic disorders, including type 2 diabetes, hyperlipidemia, *etc.* The present study investigated whether the novel polymethoxylated flavonoid pentamethylquercetin (PMQ), a member of polymethoxylated flavonoids family which is present in seabuckthorn (*Hippophae* L.) would affect adiponectin production in differentiated 3T3-L1 adipocytes. It was found that PMQ increased the adiponectin mRNA and protein expressions in adipocytes in time- and concentration-dependent manners. The PPARγ pathway plays a important roles in this effect of PMQ because blockade of PPARγ by GW9662 eliminates the PMQ-induced up-regulation of adiponectin expression. Furthermore, significant decreases of mRNA expression and secretion of TNF-α and IL-6 were also observed in PMQ-treated cells. Taken together, our study demonstrated that PMQ up-regulates adiponectin expression via a mechanism that implicates PPARγ together with TNF-α and IL-6, suggesting that PMQ might be a potential candidate for the treatment of metabolic diseases.

## 1. Introduction

Obesity or excess accumulation of visceral fat increases the likelihood of various diseases, including heart disease, type 2 diabetes, hyperlipidemia, and osteoarthritis [1]. Numerous works over the past decades have demonstrated that adipose tissue serves not only as the storage site of fat, but also an active endocrine and paracrine organ secreting adipokines, which participate in diverse metabolic processes [2]. Among the adipokines which were secreted by adipocytes, adiponectin has been particularly studied. Adiponectin is an adipocyte-derived hormone that plays a pivotal role in the regulation of lipid and glucose metabolism [3,4]. Physiologically, adiponectin stimulates fatty acid oxidation and enhances insulin-sensitivity of peripheral organs, leading to increase of glucose uptake and fatty acid oxidation in muscles [5,6]. It also exerts protective roles against chronic inflammatory and atherosclerosis [7,8,9,10]. Furthermore, several lines of evidence have indicated that plasma concentration of adiponectin is severely decreased in patients with obesity [11], type 2 diabetes, and cardiovascular diseases [12,13]. Considering the important role of adiponectin in energy metabolism, agents that up-regulate adiponectin expression and production may benefit obesity, insulin resistance, type 2 diabetes and other related metabolic diseases.

Accumulated evidence suggests that a high intake of plant foods is associated with lower risk of chronic diseases [14,15]. Recently, the flavonoids, a class of secondary metabolites extensively present in a wide range of plant foods, have received substantial attention by its manifold biological roles. Studies have shown that flavonoids protect tissues against chronic pathologies such as coronary events, cardiovascular disease mortality, and diabetes [15,16,17]. Compared with flavonoids aglycone, methoxylated flavonoids have higher intestinal absorption and are much more resistant to hepatic metabolism [18]. Furthermore, polymethoxylated flavonoids (PMFs) have been shown to alleviate metabolic disorders. For instance, nobiletin improves lipid and glucose homeostasis and modulates adipokines in fructose-induced insulin resistant hamsters [19]. Results from *in vitro* experiments also reveal that polymethoxylated flavones positively regulate adiponectin expression and production [20,21]. 

Polymethoxylated flavonoids have been shown to have better pharmacodynamic and pharmacokinetic properties compared to their polyhydroxylated analogs, however, it is hard to obtain large amounts of PMF from the natural world. Thus, we synthesized pentamethylquercetin (PMQ), a nature polymethoxylated flavonoid present in the common seabuckthorn (*Hippophae rhamnoides*) [22] and hypothesized that, in addition to myocardial protection effects [23], PMQ could regulate adiponectin expression in 3T3-L1 adipocytes. In order to elucidate its potential mechanisms, we investigated whether PPARγ, a key modulator of adiponectin production, played a role in this effect of PMQ.

## 2. Results and Discussion

### 2.1. Structure Confirmation and Identification of Synthetic PMQ

**Figure 1:**
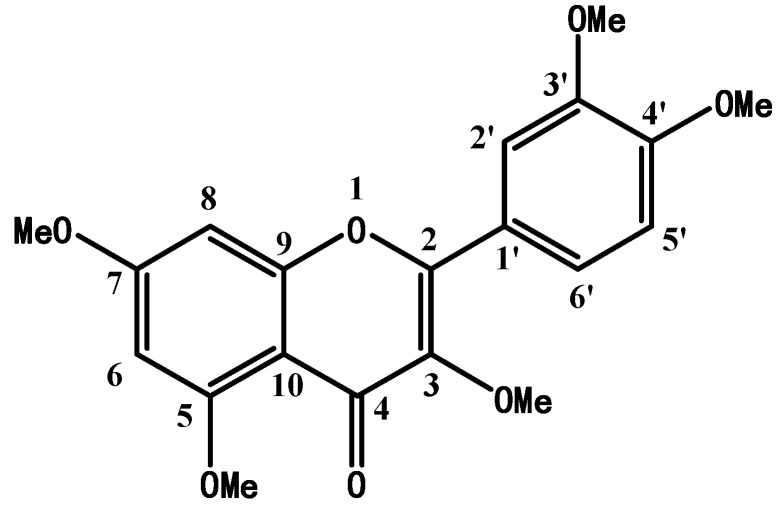
Chemical structure of pentamethylquercetin (PMQ).

The chemical structure of PMQ was showed in Figure 1. This structure was confirmed by ^1^H-NMR and ^13^C-NMR (Table 1) and was concordant with data in previous reports [24,25]. PMQ was quantified via HPLC analysis and the purity of PMQ reaches up to 99.5%. 

**Table 1:** ^1^H- (500 MHz) and ^13^C-NMR (125 MHz) data of PMQ (CDC_l3_).

Position	^13^C-NMR	^1^H-NMR
	δC	δH
1	-	-
2	152.5 sp^2^	-
3	141.1 sp^2^	-
4	173.9 sp^2^	-
5	158.7 sp^2^	-
6	95.7 sp^2^	6.343–6.348 d
7	163.8 sp^2^	-
8	92.3 sp^2^	6.502–6.508 d
9	160.9 sp^2^	-
10	109.4 sp^2^	-
1’	123.3 sp^2^	-
2’	111.1 sp^2^	7.715 s
3’	148.6 sp^2^	-
4’	150.7 sp^2^	-
5’	110.7 sp^2^	6.968–6.911 d
6’	121.5 sp^2^	7.697–7.715 d
5X-OCH_3_	55.7 sp^3^	3.847–3.989 m
	55.9 sp^3^	
	56.0 sp^3^	
	56.3 sp^3^	
	59.9 sp^3^	

### 2.2. Adipogenesis in Mouse 3T3-L1 Preadipocytes and the Effects of PMQ on Cytotoxicity and Lipid Content in Maturate Adipocyte

**Figure 2:**
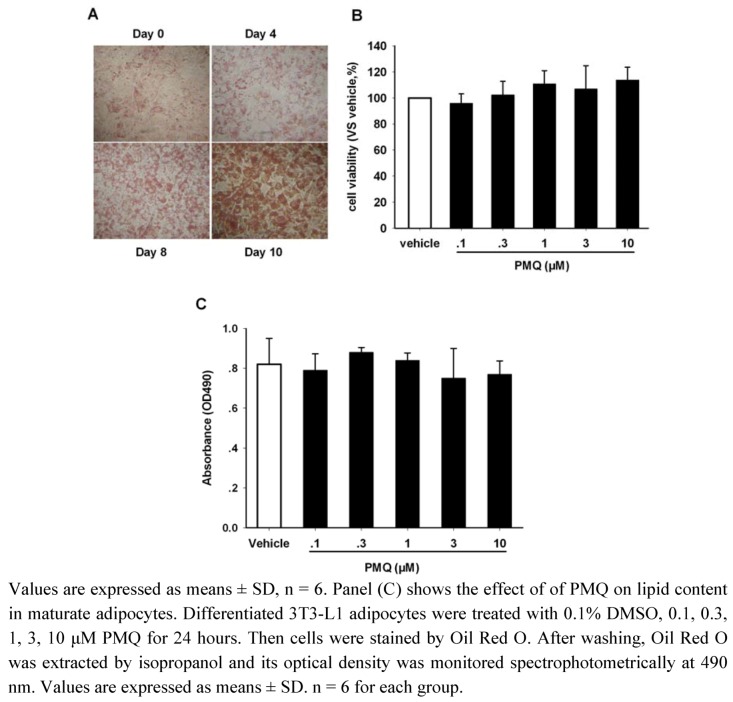
Representative morphological images of lipid uptake in cells stained by Oil Red O at day 0, 4, 8, 10 (panel A). Cells were induced to differentiate as described in the Experimental section. Panel (B) shows that incubating cells with PMQ for 24 hours did not exhibit any detrimental effect on cell viability at concentrations ranging from 0.1 to 10 μM on day 10.

Preadipocytes were cultured in medium until they reached confluency and were induced to differentiate for 10 days. Figure 2A illustrates the process during which mouse 3T3-L1 preadipocytes differentiated into mature adipocytes. Images showed cells stained by Oil Red O at day 0, 4, 8, 10. At day 10, cells were incubated with various concentrations of PMQ for 24 hours. We did not find any detrimental effect of PMQ on cell viability at concentrations ranging from 0.1 to 10 μM when incubating cells for 24 hours (P > 0.05 *vs.* vehicle) (Figure 2B). PMQ also did not show any effects on lipid content in maturate adipocytes (P > 0.05 *vs.* vehicle) (Figure 2C).

### 2.3. Effect of PMQ on Adiponectin Expression in 3T3-L1 Adipocytes

**Figure 3:**
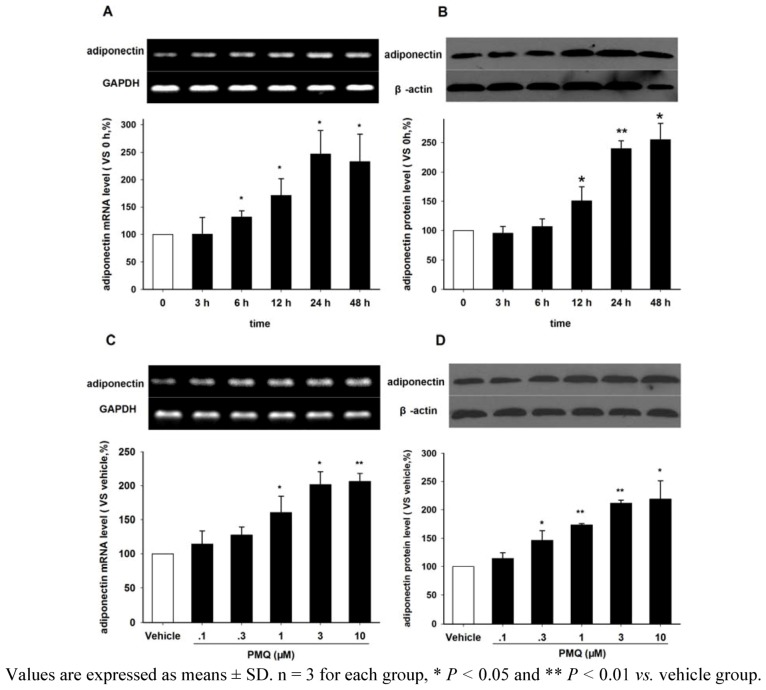
Effects of PMQ on adiponectin mRNA and protein expression in 3T3-L1 adipocytes. **A.** RT-PCR images and semi-quantatative values of adiponectin mRNA level in 3T3-L1 adipocytes treated with PMQ (3 μM) for 0, 3, 6, 12, 24, 48 hours. **B.** Western blot images and semi-quantatative values of adiponectin protein level in 3T3-L1 adipocytes. **C.** RT-PCR images and semi-quantatative values of adiponectin mRNA level in 3T3-L1 adipocytes treated with different concentrations of PMQ or vehicle for 24 hours. **D.** Western blot images and semi-quantatative value of adiponectin protein level in 3T3-L1 adipocytes.

Figure 3 show the effects of PMQ on adiponectin mRNA and protein expressions in differentiated 3T3-L1 adipocytes. We found that PMQ increased adiponectin mRNA expression in a time-dependent manner (Figure 3A This increase started at 6 hours after PMQ treatment and reached a peak level at 24 hours. Western blot analysis with an anti-adiponectin antibody revealed that the level of adiponectin protein significantly increased at 12 hours, but not at 3 or 6 hours after PMQ treatment, compared to 0 hours controls (Figure 3B). Figure 3C showed representative RT-PCR results of differentiated 3T3-L1 cells treated with various concentrations of PMQ (0.1, 0.3, 1, 3 and 10 μM) for 24 hours, with 0.1% DMSO treated cells as controls. It was found that PMQ increased adiponectin mRNA (P < 0.05) expression at the concentrations of 1, 3 and 10 μM, but not at 0.1 and 0.3 μM (Figure 3C. Results from Western blot analysis also confirmed the dose-dependent change in adiponectin protein expression after PMQ treatment (Figure 3D).

### 2.4. PMQ Regulates Adiponectin Expression via PPARγ Pathway

**Figure 4:**
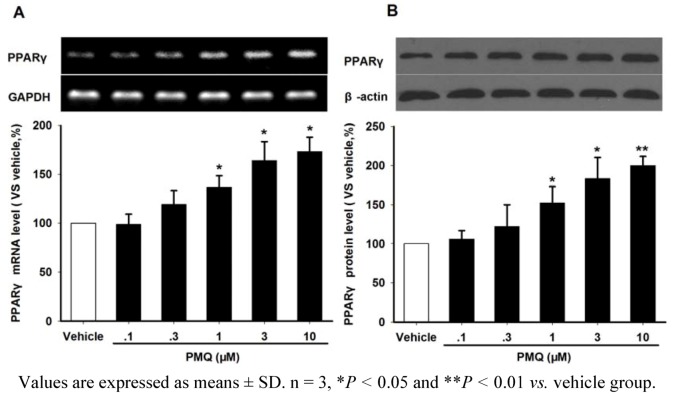
Effects of PMQ on PPARγ mRNA and protein expression in 3T3-L1 adipocytes. A. RT-PCR images and semi-quantatative values of PPARγ in 3T3-L1 adipocytes treated with different concentrations of PMQ or vehicle. B. Western blot images and semi-quantatative values of PPARγ protein level in 3T3-L1 adipocytes.

PPARγ is a pivotal modulator for adiponectin production in adipocytes. In the current study, we investigated whether PPARγ pathway were involved in the effect of PMQ on adiponectin regulation in 3T3-L1 adipocytes. RT-PCR and Western blot analysis revealed that PMQ significantly increased the PPARγ mRNA and protein expressions in adipocytes at concentrations above 1 μM (Figure 4). 

**Figure 5:**
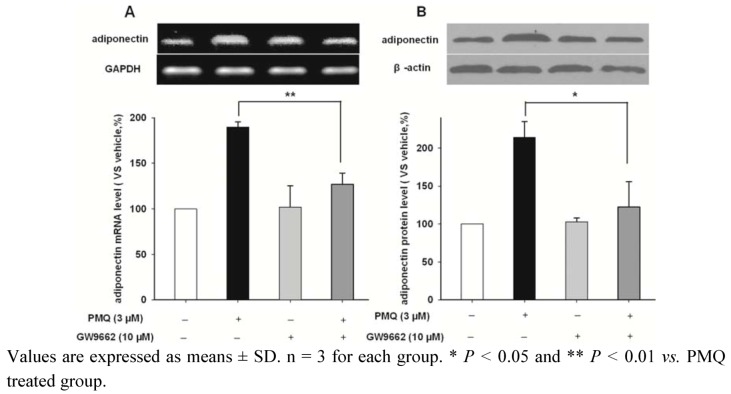
Effects of PPARγ inhibitor GW9662 on PMQ-induced adiponectin expression in 3T3-L1 adipocytes. **A.** RT-PCR images and semi-quantatative values of adiponectin in 3T3-L1 adipocytes treated with PMQ (3 μM), either with or without GW9662 (10 μM.). **B.** Western blot images and semi-quantatative values of adiponectin protein level in 3T3-L1 adipocytes.

GW9662, an inhibitor of PPARγ, was applied in order to corroborate this observation. Figure 5 shows the mRNA and protein levels of adiponectin in 3T3-L1 adipocytes treated with vehicle, PMQ, GW9662 and GW9662 + PMQ. We showed that treating cells with 10 μM GW9662 alone had no effect on adiponectin expression. However, GW9662 could significantly attenuate PMQ-induced increase of adiponectin in mRNA and protein levels (Figure 5). These results indicated that PPARγ pathway mediates the increase of adiponectin expression induced by PMQ. 

### 2.5. Effects of PMQ on Expression and Secretion of TNF-α and IL-6

**Figure 6:**
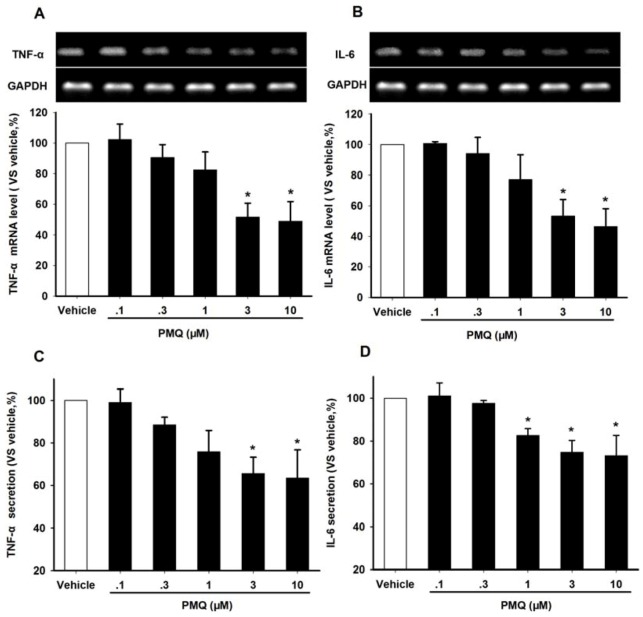
Effects of PMQ on TNF-α and IL-6 mRNA expression and secretion in 3T3-L1 adipocytes. **A.** RT-PCR images and semi-quantatative values of TNF-α in 3T3-L1 adipocytes treated with different concentrations of PMQ or vehicle. **B.** RT-PCR images and semi-quantatative values of IL-6. Values are expressed as means ± SD. n = 3 for each group. * *P＜* 0.05, ** *P＜* 0.01 *vs.* vehicle. **C.** Quantitative value of TNF-α secretion in culture medium by ELISA kits. D. IL-6 secretion in culture medium. Data are expressed as % (means ± SD) of three separate samples each performed in duplicate. n = 3 for each group, *P＜ 0.05 *vs.* vehicle group.

Both TNF-α and IL-6 are negative regulators of adiponectin expression [26,27]. Here we investigated whether PMQ also modulated the expressions of TNF-α and IL-6 in 3T3-L1 adipocytes. RT-PCR analysis revealed that PMQ decreased mRNA expression of TNF-α and IL-6 in a concentration-dependent manner, with the peak change at 3 and 10 μM (Figure 6A and B). Results from ELISA detection also showed that secretion of TNF-α and IL-6 in culture medium decreased significantly in cells treated with PMQ. These data suggested that TNF-α and IL-6 were also involved in PMQ-induced adiponectin expression (Figure 6C and D).

In the present study, we have demonstrated that the synthetic polymethoxylated flavonoid pentamethylquercetin (PMQ) increased adiponectin expression in differentiated 3T3-L1 adipocytes in time- and dose-dependent manners. This effect is mediated by PPARγ pathway because blockade of PPARγ by GW9662 eliminates PMQ-induced increase of adiponectin production. Furthermore, TNF-α and IL-6 are also involved in PMQ-mediated up-regulation of adiponectin expression. Considering the important role of adiponectin in energy metabolism, we postulate that PMQ treatment may benefit obesity, insulin resistance, type 2 diabetes and other related metabolic diseases.

Obesity is a pandemic health problem in both developed and developing countries, which increases the risks of many common diseases, including coronary artery disease (CAD), type 2 diabetes, hypertension, gall bladder disease, and osteoarthritis [28]. Plasma adiponectin is found to be lower in obese human subjects, and reducing body weight is helpful for improving plasma adiponectin level [11,29]. Furthermore, the steady state mRNA of adiponectin was found to be down-regulated in ob/ob mice, suggesting the relationship between obesity and adiponectin expression [4]. Correspondingly, deletion of the mouse adiponectin gene, which induces low plasma adiponectin levels, is associated with metabolic syndrome including insulin-resistant diabetes and atherosclerotic disease [30]. Considering its extensive beneficial effects in diabetes, atherosclerotic, inflammation etc, adiponectin is deemed as a new target for treatment of metabolic syndrome. Some pharmaceutical companies have attempted syntheses of adiponectin, however, no synthetic adiponectin with *in vivo* bioactivity has been obtained so far. Therefore, it is of great clinical significance to search a new medicine which can stimulate the production of endogenous adiponectin. In this study, we show that polymethoxylated flavonoid pentamethylquercetin (PMQ) increase adiponectin mRNA expression and intracellular content in 3T3-L1 adipocytes, suggesting that PMQ could be a potential candidate for the treatment of obesity, metabolic syndrome, and type 2 diabetes.

PPARγ is a type II nuclear receptor that in humans is encoded by the PPARγ gene and plays a significant role in mediating insulin sensitivity [31]. It regulates fatty acid storage and glucose metabolism by regulating the transcription of many adipocyte-specific genes to stimulate lipid uptake and adipogenesis in fat cells. A large body of evidence confirms that PPARγ, mainly expressed in adipose tissue, is a positive regulator of adiponectin gene expression. For instance, adipose-tissue-specific deletion of the PPARγ gene exhibits a decay of plasma adiponectin levels in mice [32]. Thiazolidinediones (TZDs), a well-known PPAR*γ* agonist, stimulates adiponectin transcription both *in vitro* and *in vivo* [33,34]. In addition, both human [35] and mouse [36] adiponectin promoters contain a putative PPAR-responsive element (PPRE) and point mutations at this site lead to reduced basal and TZD-induced adiponectin promoter transactivation [35]. These suggest that PPARγ may enhance cellular adiponectin level by acting directly on its promoter. Herein, for the first time, we report that PMQ enhances PPARγ mRNA and protein levels, and PPARγ antagonist GW9662 abolishs the increase of adiponectin mRNA and protein level induced by PMQ. Although the exact mechanism through which PMQ modulates PPARγ remains unknown, these data still strongly suggest that PMQ regulates adiponectin production via a PPARγ-dependent manner. 

TNF-α and IL-6 are negative regulators of adiponectin expression. Serum concentration of IL-6 is elevated in insulin-resistant states such as obesity [37,38,39], impaired glucose tolerance [40] and type 2 diabetes [41,42]. Furthermore, IL-6 treatment suppresses mRNA levels and secretion of adiponectin in 3T3-L1 adipocytes [26], suggesting a negative role of IL-6 in the modulation of adiponectin levels and insulin sensitivity. Previous studies have shown that TNF-α down-regulates adiponectin gene expression in adipocytes by suppressing the expression of activators involved in promoting adiponectin gene expression, such as PPARγ [43], C/EBP [44,45]. This suppressive effect of TNF-α on adiponectin transcription may be mediated by JNK (c-Jun N-terminal kinase), which has been shown to phosphorylate PPARγ and decrease its DNA-binding activity [27,46]. Our present study showed that PMQ treatment significantly decreased TNF-α and IL-6 mRNA expressions and secretions in 3T3-L1 adipocytes, suggested that TNF-α and IL-6 may be involved in PMQ-induced adiponectin expression. As mentioned above that TNF-α suppresses the expression of PPARγ, it suggests that PMQ might enhance adiponectin expression through decreasing the repressive effect of TNF-α on PPARγ pathway. 

## 3. Experimental

### 3.1. Chemicals

Quercetin was obtained from Sewoer Industry and Trade Co., Ltd. (Hangzhou, China). Acetone, sodium hydroxide and anhydrous potassium were obtained from Sinopharm Chemical Reagent Co., Ltd. (Beijing, China). Dimethyl sulfate was obtained from Kelong Chemical Reagent Factory (Chengdu, China). Isobutyl-3-methylxanthine (IBMX), GW9662, wortmannin, dexamethasone, insulin and Oil Red O were obtained from Sigma-Aldrich Chemical Co. (St Louis, MO, USA). Dulbecco’s modified Eagle’s medium (DMEM) was purchased from Gibco (Invitrogen, Carlsbad, CA, USA) and fetal bovine serum (FBS) was purchased from Sijiqing (Hangzhou, China). Anti-PPARγ rabbit IgG , anti-β-actin mouse IgG and anti-adiponectin goat IgG were obtained from Santa Cruz Biotechnologies (Santa Cruz, CA, USA). HRP-linked anti-rabbit IgG, anti-mouse IgG and anti-goat IgG were obtained from Pierce (Thermo Scientific, Rockford, IL, USA). ELISA Kits for TNF-α and IL-6 were obtained from R&D Systems (Minneapolis, MN, USA).

### 3.2. Preparation of Pentamethylquercetin

Firstly, we converted quercetin into 3,7,3’,4’-tetramethylquercetin by heating to 50 °C with acetone, dimethyl sulfate and anhydrous potassium carbonate for 7 hours. Pentamethylquercetin (PMQ) is obtained through the agency of additional dimethyl sulfate and sodium hydroxide added after 2 hours.The structure of the end product was confirmed by mass spectrometry, ^1^H-NMR and ^13^C-NMR. The purity of PMQ was examined by high performance liquid chromatography and reached 99.5% after being recrystallized twice from anhydrous alcohol.

### 3.3. Cell Culture and Treatment

Mouse 3T3-L1 preadipocytes from American Type Culture Collection (Manassas, VA, USA), were cultured in DMEM containing 25 mM glucose supplemented with 10% bovine serum at 37 °C in a humidified atmosphere with 5% CO_2_. Cells were subcultured every 3–4 days at approximately 90% confluence and cells were plated at a density that allowed them to reach confluence in 2 days. Completely confluent plates were incubated in DMEM containing 10% FBS with 0.5 μM 3-isobutyl-1-methylxanthine (IBMX), 0.25 μM dexamethasone and 10 μg/mL insulin.Two days after incubation, the medium was replaced with DMEM containing 10 μg/ml insulin. Thereafter, cells were maintained in the original propagation DMEM with medium changes every 2–3 days, the differentiation was completed at day 10. At day 10, cells were incubated with various concentrations of PMQ or vehicle for 24 hours. 3T3-L1 cells differentiated for 10 days were incubated for 24 hours with PMQ (3 μM.), either with or without GW9662 (10 μM.).

### 3.4. Oil Red O Staining

Adipocyte differentiation was monitored by measurement of lipid accumulation through staining of neutral fats and cholesterol esters with Oil Red O. Cells were fixed with 4% formalin for 5 min, and stained for 10 min in freshly diluted Oil Red O solution (0.5%). After washing, Oil Red O was extracted by isopropanol and its optical density was monitored spectrophotometrically at 490 nm.

### 3.5. Cell Viability Assay

Cell viability was determined by the 3-(4,5-dimethylthiazol-2-yl)-2,5-diphenyltetrazolium bromide (MTT) assay. 3T3-L1 cells were plated into 96-well until they reached confluency and quiescent. Then the cells were induced to differentiate for 10 days. The maturate adipocytes were switched to a culture medium containing PMQ for 24 h and then PBS-buffered MTT (20 μL, 5 mg/mL) solution was added to each well and the plates were incubated at 37 °C for additional 4 h. The medium was then removed, and 100 μL/well DMSO was applied to each well to dissolve the purple formazan crystals. The plates were read (wavelengths: test, 492 nm; reference, 570, 630 nm) using a microplate spectrophotometer (Tecan, Genois Ltd, Männedorf, Switzerland). Results were standardized using vehicle group values.

### 3.6. Reverse Transcription-Polymerase Chain Reaction

Total RNA of 3T3-L1 cells was extracted using Trizol reagent (Invitrogen) following the manufacturer’s instruction. Reverse transcription (RT) was performed with the RT system (Promega) protocol in a 50 μL reaction mixture using oligo(dT)18 primers. After the RT procedure, the reaction mixture (cDNA) was used for polymerase chain reaction (PCR). Primers of corresponding genes are shown in Table 2. The reaction was conducted with the following protocol: initial denaturing of the template for 5 minutes at 94 °C followed by 40 repeating cycles of denaturing for 30 seconds at 94 °C, annealing for 30 seconds at 50–63 °C, extension for 1 minute at 72 °C, and a final elongation at 72 °C for 10 minutes. The PCR products were size-fractionated by 2.5% agarose gel electrophoresis and visualized by SYBR Green I (Solarbio) staining. 

**Table 2:** Mouse gene-specific primers for RT-PCR.

Gene	Sequences (5’ -3’)	Accession No	Length (bp)
PPARγ	Forward: ACTGCCTATGAGCACTTCAC	NM_011146	448
	Reverse: CAATCGGATGGTTCTTCGGA		
Adiponectin	Forward: GGGTGAGACAGGAGATGTTGGAATG	NM_009605	478
	Reverse: GCCAGTAAATGTAGAGTCGTTGACG		
IL-6	Forward: AGTTGCCTTCTTGGGACTGA	NM_031168	521
	Reverse: GCCACTCCTTCTGTGACTCC		
TNF-α	Forward: TGGAGTCATTGCTCTGTGAAGGGA	NM_013693	250
	Reverse: AGTCCTTGATGGTGGTGCATGAGA		
GAPDH	Forward: GACAAAATGGTGAAGGTCGGTG	NM_008084	256
	Reverse: TGATGTTAGTGGGGTCTCGCTC		

### 3.7. Western Blotting

Cells were harvested and lysed in RIPA buffer containing 150 mM NaCl, 1% NP-40, 0.5% sodium deoxycholate, 0.1% sodium dodecyl sulfate (SDS), 50 mM Tris–HCl (pH 7.4), 50 mM glycerophosphate, 20 mM NaF, 20 mM EGTA, 1 mM DTT, 1 mM Na_3_VO_4_, 1 mM phenylmethylsulfonyl fluoride, and protease inhibitors. Total cell proteins were separated by 12% SDS–polyacrylamide gel electrophoresis, transferred to a polyvinylidene fluoride membrane, and then hybridized with primary antibodies (diluted 1:400–1,000) overnight at 4 °C. After incubation with horseradish-peroxidase-conjugated secondary antibody (diluted 1:3,000) for 1 h at room temperature, immunoreactive proteins were visualized using an enhanced chemiluminescent reagent (Amersham Pharmacia Biotech, Piscataway, NJ, USA). 

### 3.8. ELISIA

Secretion of TNF-α and IL-6 was determined by the ELISA Quantikine Mouse TNF-α and IL-6 Immunoassay (R&D Systems, Minneapolis, MN), according to the manufacturer's instruction.

### 3.9. Statistical Analysis

Data are expressed as means ± SD. One-way ANOVA followed by Student’s two-tailed unpaired *t*-test was used for the statistical analysis by SPSS 11.0 software. Values of P < 0.05 were considered to indicate statistical significance.

## 4. Conclusions

The data herein presented demonstrates that pentamethylquercetin (PMQ), a synthetic polymethoxylated flavonoid, improved adiponectin mRNA expression and protein levels in 3T3-L1 adipocytes. This effect is related to enhanced PPARγ function and down-regulated TNF-α and IL-6. Our study raises a novel finding that PMQ could be a promising new candidate for the treatment of metabolic diseases in the future. 
